# The role of self-compassion in adults with dyslexia

**DOI:** 10.1007/s11881-025-00327-0

**Published:** 2025-04-16

**Authors:** Georgia Niolaki, Alexandra-Iuliana Negoita, Aris Terzopoulos, Jackie Masterson

**Affiliations:** 1 University of Birmingham, Birmingham, UK; 2 Birmingham City University, Birmingham, UK; 3 Coventry University, Coventry, UK; 4 Institute of Education, UCL, London, UK

**Keywords:** Adulthood, Anxiety, Dyslexia, Overidentification, Self-compassion, Self-efficacy, Self-esteem

## Abstract

The emotional aspect of dyslexia has recently received more attention. A growing body of literature highlights the links between dyslexia and self-perception (such as self-esteem and self-efficacy) and psychopathology (such as anxiety). However, there is no research on self-compassion in adults with dyslexia. The current study aimed to examine the role of self-compassion in relation to self-esteem, self-efficacy and anxiety in adults with dyslexia. We investigated whether facets of self-compassion have distinct roles within these relationships. We also aimed to identify whether self-compassion mediates the association of self-esteem and self-efficacy with dyslexia and anxiety. Findings are reported from 100 adults with dyslexia who took part in an online survey involving measures of self-compassion, self-esteem and self-efficacy, and anxiety. Self-compassion was related to the other measures and was found to mediate the association of anxiety with self-esteem and self-efficacy, which has not been reported before in adults with dyslexia.

According to the Diagnostic and Statistical Manual of Mental Disorders – Fifth Edition (American Psychiatric Association (APA), 2022), dyslexia is a neurodevelopmental specific learning disorder (SpLD) that causes, among others, difficulties in learning to read, spell and write. Dyslexia is a highly heritable disorder that can significantly impact individuals’ lives (Gialluisi et al., 2020; Snowling et al., 2020). In the UK and worldwide, around one in 10 individuals have dyslexia (Shaywitz et al., 2021). Hence, it is considered to be the most common SpLD, and as it is characterised as a hidden disability, many individuals remain undiagnosed (Snowling et al., 2020). This suggests that the number of individuals with dyslexia could be much higher. Even so, the UK government reported that dyslexia is one of the most common special educational needs (SEN) difficulties and the one that receives the most support in schools (Department for Education (DfE), 2021). Although dyslexia in school age has been thoroughly investigated, less is known about adults with dyslexia and the emotional obstacles they must overcome to succeed and reach their full potential. Recent publications highlight the importance of supporting students with literacy difficulties as they transition into higher education and beyond (Abbott-Jones, 2021). They also emphasise the need to recognise and address these students’ emotional needs in addition to their cognitive challenges (e.g., Carroll & Iles, 2006; Niolaki et al., 2020). Similarly, the British Dyslexia Association (BDA) (2021) argues for the necessity of additional efforts from employers to provide dyslexic employees with a suitable, adjusted environment that can help them succeed (British Dyslexia Association (BDA), n.d.).

Although dyslexia, by definition, is not directly related to emotional difficulties (American Psychiatric Association (APA), 2022; Snowling, 2019), the emotional aspect of dyslexic difficulties is receiving more attention. There is a growing body of literature that highlights the links between dyslexic difficulties with negative self-perception (such as low self-esteem and self-efficacy) and psychopathology (such as anxiety) throughout the lifespan (Burden, 2008; de Beer et al., 2014; Livingston et al., 2018; Gibby-Leversuch et al., 2019). Self-perception refers to how individuals think and feel about themselves and incorporates terms such as self-esteem and self-efficacy (McArthur et al., 2020). Self-esteem is defined by the feelings one has towards the self as a result of self-evaluation processes (Burden, 2008). Self-efficacy is defined by how people feel about their ability to perform successfully in a given task (Chen et al., 2001). Self-efficacy and self-esteem are related concepts, both involving self-evaluation. However, self-efficacy involves the evaluation of more specific situations rather than one’s broader abilities (Chen et al., 2001). Anxiety is characterised by feelings of unease, such as fear or worry, which can be mild or more severe and negatively impact overall well-being (APA, 2022). Due to their association with dyslexia, self-esteem, self-efficacy and anxiety have all been included as variables in the current study.

Despite the evidence highlighting the emotional implications of dyslexia, mental health is a frequently overlooked aspect when identifying and working with individuals with dyslexia (Ryder & Norwich, 2018). Hence, it is important to investigate mechanisms that can minimise this impact. Based on findings from studies with a non-dyslexic population (Dupasquier et al., 2020; Fong & Loi, 2016; MacBeth & Gumley, 2012; Neff, 2003a; Zhang et al., 2016), self-compassion is proposed to be one such factor that can mediate the effects of negative emotional outcomes. Self-compassion is broadly defined as a caring attitude towards the self, being open and non-judgemental of personal flaws and involving a willingness to alleviate suffering with kindness (Neff, 2003a). Neff (2003b) identified three facets of self-compassion, each within two extreme endpoints of a continuum. The first facet is self-kindness vs. self-judgement; the former represents the extent to which one is kind and understanding with oneself, and the latter is being harsh, judgemental and self-criticising. The second is common humanity vs. isolation; the former refers to seeing one’s experiences as part of the larger human experience, and the latter sees oneself as separate and isolated. The final is mindfulness vs. over-identification; the first concentrates on holding one’s painful thoughts and feelings in balance, and the latter on over-identifying with thoughts and feelings.

The current study aimed to investigate the role of self-compassion in adults with dyslexia and how this relates to self-esteem, self-efficacy and anxiety, which have not yet been investigated using all these variables simultaneously in adults with dyslexia, as demonstrated in the following parts of our literature review. Prior research indicates that self-compassion could have a significant effect on the relationship between negative emotions and self-esteem and self-efficacy in other neurotypical or neurodiverse populations (Dupasquier et al., 2020; Fong & Loi, 2016; MacBeth & Gumley, 2012; Neff, 2003a; Zhang et al., 2016). We used the biopsychosocial model (Engel, 1977) as a lens to conceptualise the emotional challenges nested in dyslexia (Doyle, 2020) and to understand the counselling needs of dyslexic individuals (Elftorp & Hearne, 2020). The biopsychosocial model (Engel, 1997) views difficulties influenced by biological, psychological and social factors, which, yarned together, provide a comprehensive understanding of individuals’ experiences. The model views dyslexia as a neurological developmental difficulty; however, it also acknowledges that psychological and social experiences affect people’s perception of the dyslexic challenges (Doyle, 2020; Elftorp & Hearne, 2020).

Burden (2008) and Mugnaini et al. (2009) were the first to review the literature on the emotional impact of dyslexia. Burden’s (2008) results highlighted that whilst dyslexia was associated with negative self-perception in children, such as low self-efficacy and self-esteem, these feelings are dependent upon many factors, such as academic progress and social support. Mugnaini et al. (2009) reviewed eleven studies published between 2000 and 2008 and found moderate and large effects (ranging from 3 to 0.3), demonstrating that being dyslexic is associated with risk of psychological discomfort, particularly internalising issues, leading to anxiety and depression. Whilst these reviews generate some understanding of the emotional implications of dyslexia, they do not produce a detailed understanding of how these outcomes relate to one another.

Building on Burden’s (2008) findings, Gibby-Leversuch et al. (2019) included more recent studies in their systematic review covering findings from nineteen papers with 1122 participants. This review found predominantly medium-sized effects on the association between learning difficulties, including dyslexia, and increased risk of developing negative self-perception as a learner, but added that this does not impact the overall self-worth of the individuals. This could mean that dyslexia might influence global self-perception (such as overall self-efficacy) differently than domain-specific self-perception (such as reading and writing self-efficacy). However, Burden (2008) and Gibby-Leversuch et al. (2019) only included studies with children and young people. The potential long-term effects of this in adulthood also need to be examined.

Livingston et al. (2018) provided a more comprehensive overview of the emotional impact of dyslexia concerning self-esteem and anxiety. Ninety-seven quantitative and qualitative papers published between 1980 and 2018, involving samples from childhood to adulthood, were included in their study. The findings indicate that dyslexia has a detrimental impact on self-evaluation and feelings of inadequacy, affecting self-esteem, behaviour, motivation and emotional well-being. Although the authors included studies of dyslexia from childhood to adulthood, the majority of the studies only included children. This signposts a lack of and a need for studies focusing on adult experiences and outcomes.

Most of the previous research conducted with adults has focused on concepts of self-perception. Low self-esteem is consistently reported in the literature as being linked with dyslexia (Burden, 2008; Carawan et al., 2015; Dåderman et al., 2014; Livingston et al., 2018; McNulty, 2003; Mugnaini et al., 2009; Nalavany et al., 2010; Nalavany et al., 2011; Riddick et al., 1999). Self-esteem has a fundamental role in psychological adjustment; thus, low self-esteem increases the risk of developing feelings of unworthiness and negative emotions (Marshall et al., 2015; Terras et al., 2009). In their review, Livingston et al. (2018) suggested that feelings of inadequacy cause low self-esteem in the dyslexic population. Hence, self-esteem is linked with psychological well-being in adults with dyslexia (Dåderman et al., 2014; Terras et al., 2009).

Self-efficacy is closely related to self-esteem. More negative or uncomfortable emotions emanating from living with dyslexia were found to predict lower levels of total work self‐efficacy, work attributes, work competency and work anxiety over and beyond background variables (Nalavany et al., 2017). This means that the adverse outcomes of dyslexia are experienced similarly regardless of one’s background.

The above findings are strengthened by de Beer et al.’s (2014) systematic review which included 33 quantitative and qualitative papers published after 1995. De Beer et al. (2014) did not aim to look at the concept of self-efficacy and self-esteem specifically. However, they aimed to identify factors influencing work participation in a dyslexic adult population. They found that the participants felt that difficulties associated with dyslexia (reading and spelling) negatively influenced work participation and caused feelings of inadequacy when considering literacy-related tasks. The total number of participants in qualitative studies was 256; in quantitative studies, it exceeded 17,000, making the findings highly generalisable. This demonstrates that negative self-evaluation can negatively impact career performance. It is, therefore, essential to identify factors that can become mediators and consequently boost the individuals’ self-esteem.

In some studies, dyslexia and also negative experiences or feelings caused by dyslexia were associated with anxiety (Bergey et al., 2015; Carroll & Iles, 2006; McNulty, 2003) and some authors suggest that these links are more evident in academic anxiety rather than more global anxiety (Bergey et al., 2015; Carroll & Iles, 2006; Elgendi et al., 2021; Jordan et al., 2014). Nevertheless, quantitative studies which compared levels of anxiety between dyslexic and non-dyslexic populations found significant but weakly moderate (*d* = 0.41 in Bergey et al., 2015; *η*_*p*_^*2*^ = 0.05 in Elgendi et al., 2021) and strong (*η*_*p*_^*2*^ = 0.40 in Carroll & Iles, 2006) effect differences for anxiety scores. In a recent meta-analytic study, Vieira et al. (2024) examined anxiety in individuals with reading difficulties. The authors found that in the cluster variable—including anxiety and other internalizing problems—the overall effect size was moderate and significant (Hedges’* g* = − 0.54). When examining different subcomponents of anxiety that could influence this relationship, separation anxiety had the greatest impact compared to other forms of anxiety, such as academic and generalized anxiety. However, the researchers did not explore state and trait anxiety, which we included in our study, and their sample consisted of children and adolescents rather than only adults.

MacBeth and Gumley (2012) found a large effect size in a meta-analysis exploring the association between self-compassion and anxiety (*Z* = − 34.02; *p* < 0.0001). However, this study did not include individuals with dyslexia. In line with the self-compassion theory (Neff, 2003b), this study confirmed that self-compassion can effectively reduce the negative impact of anxiety on well-being. Dupasquier et al. (2020) further supported this with a neurotypical population. They suggested that self-compassion relates positively to positive affect, higher well-being and lower distress and negatively to negative affect and stress, similar to findings from other studies (Fong & Loi, 2016; Zhang et al., 2016). It could then be argued that self-compassion might decrease the effects of negative self-perception on anxiety in individuals with dyslexia.

Conclusions about the role of different facets of self-compassion cannot be drawn, as the self-compassion scale (SCS; Neff, 2003a) is typically reported as a single overall score in the empirical literature rather than through its subscales. MacBeth and Gumley (2012) have suggested that examining these subscales could provide valuable insights. Results of a study by Zhang et al. (2016) with 208 participants also indicated that there could be a different role for distinct facets of self-compassion, as their analysis showed that positive aspects of self-compassion did not correlate significantly, or only weakly, with learning stress and negative affect, whilst negative facets all correlated significantly with learning stress and negative affect. Scores for the common humanity sub-scale seemed the least associated with other variables. It might be that specific mechanisms better mediate the relationship between emotional outcomes of dyslexia than others. Suppose potential multicollinearity issues between the sub-scales of self-compassion can be overcome. In that case, it is worth investigating the role of different facets individually. In the current study, we aimed to investigate the role of each component in mediating the negative effects of self-perception on anxiety.

## The current study

Dyslexia can have a negative emotional impact throughout the lifespan (Burden, 2008; Gibby-Leversuch et al., 2019; Livingston et al*.,* 2018). However, this has only been studied in relation to self-esteem (Dåderman et al., 2014) and self-efficacy (Nalavany et al., 2017) in adults. Hence, it is currently unknown how self-compassion affects adults with dyslexia or the role of self-compassion as a potential mediator of the well-reported relationship between low self-esteem and self-efficacy with anxiety in individuals with dyslexia. Nevertheless, research has indicated that self-compassion can mediate the influence of low self-esteem on mental health (Neff, 2003a, 2016b; Neff, 2020) and that it is associated with psychological well-being (Fong & Loi, 2016). As such, the current study aimed to examine the role of self-compassion in adults with dyslexia and investigate whether aspects of self-compassion can mediate the effect of low self-perception (self-esteem and self-efficacy) on anxiety.

The present study aimed to address three research questions. The first concerned whether self-compassion, self-perception (self-esteem and self-efficacy) and anxiety are related in adults with dyslexia. Based on findings from previous studies (Dupasquier et al., 2020; Fong & Loi, 2016; Neff, 2003a; Zhang et al., 2016), we hypothesised that positive aspects of self-compassion (self-kindness, mindfulness and common humanity) would be positively associated with high self-esteem and self-efficacy. Equally, negative facets of self-compassion (self-judgement, isolation and over-identification) would be associated with anxiety and low self-esteem and self-efficacy. Self-esteem was expected to be positively associated with self-efficacy, whilst both these were expected to be negatively associated with anxiety. The second research question asked what the role of self-compassion in the association of self-efficacy and self-esteem with anxiety in adults with dyslexia is. Based on previous research examining the role of self-compassion (Neff, 2003a; Neff, 2016b; Neff, 2020; Fong & Loi, 2016), we expected that self-compassion could be a significant mediator of the effects of self-esteem and self-efficacy on anxiety. The third research question asked whether different facets of self-compassion have distinct roles as mediators in the effects of self-esteem and self-efficacy on anxiety.

## Method

### Participants

We invited adults to respond to a survey in order to identify those who fit the criteria for taking part in the study. One hundred and two adults with dyslexia responded to the survey. To take part, participants had to have a formal diagnosis of dyslexia or else to self-identify as having dyslexia. They also had to be over the age of 18. Two survey respondents were excluded as they did not confirm they were over the age of 18. In total, there were 78 participants with a formal diagnosis of dyslexia and 22 participants who self-reported as having dyslexia. Having self-reported dyslexics is not unexpected in an adult sample. Snowling et al. (2012) demonstrated that self-reported dyslexia can be a valid way to identify dyslexic challenges (also see O’Dwyer et al., 2024). Before treating the sample as a whole, we carried out independent sample *t*-tests that confirmed that in all critical variables (i.e., scores for self-esteem, self-efficacy, self-compassion and anxiety), there were no significant differences between the two groups (all *p*s > 0.05, both one-tailed and two-tailed comparisons).

In terms of gender, there were 69 female participants. Although this balance may be considered anomalous based on research evidence that indicates a prevalence of males rather than females with dyslexia, there is also the opposite evidence suggesting that the higher male ratio may be due to referral and conduct biases (Arms et al., 2008; Shaywitz et al., 1990). Characteristics of the participants are presented in Table 1.

**Table 1:** Participant characteristics

Variable	Number of participants	Category
Age	13 26 35 19 6 1	18–24 25–34 35–44 45–54 55–64 Over 65
Gender	69 28 3	Female Male Not specified
Diagnosis of dyslexia	78 22	Formal diagnosis Self-identified
Age of diagnosis (*N* = 68)	19 17 15 17	5–11 12–18 19–24 Over 25
Ethnicity	82 5 3 3 2 5	White Mixed/Multiple Asian/British Asian Black, African, Caribbean or Black British Other Unknown
Education	37 18 9 9 7 6 14	Bachelor’s degree Master’s degree College Doctorate Vocational training No formal education Unknown
Language	86	English as a first language
Narration	100	Without narration

All characteristics are representative of the time of data collection and of those who completed all sections and met all inclusion criteria (*N *= 100).* Narration*: Needing someone to read the questions aloud (option provided)

Participants were recruited by advertising the research to British Dyslexia Association members, to local dyslexia associations and on social media (Twitter, LinkedIn and Facebook).

## Materials

### Self-esteem

Rosenberg’s self-esteem scale (SES; Rosenberg, 1965) was employed to assess participants’ beliefs about their self-worth. The scale has 10 items with a four-point Likert scale (with 1 representing ‘strongly agree’ and 4 representing ‘strongly disagree’). The measure includes statements assessing competence (e.g., *‘I can accomplish tasks as well as most people’*) and self-liking (e.g., *‘I have a positive attitude toward myself’*) (Schmitt & Allik, 2005). Overall, if someone receives a score between 0 to 14, this is considered low self-esteem, 15–25 is considered moderate self-esteem, and 26 and above is high self-esteem (García et al., 2019). Research has shown that the scale demonstrates strong convergent and discriminant validity based on data from 53 countries (Schmitt & Allik, 2005). Moreover, Torrey et al. (2000) reported high internal consistency and test–retest reliability of the scale over 2 weeks (*α* = 0.87). In the present study, sub-score totals, as well as an overall score, were calculated with some items reverse scored, as instructed by the developer (Rosenberg, 1965). Cronbach’s *α* is reported to be 0.83 in the original study (Rosenberg, 1965). In the present study, the total scale reported a Cronbach’s *α* coefficient of 0.77.

### Self-efficacy

To assess how participants felt about their ability to perform successfully in different tasks and situations, the general self-efficacy scale (GSES; Chen et al., 2001) was used. The scale has eight items with a five-point Likert scale (where 1 represents ‘strongly agree’ and 5 ‘strongly disagree’). Scores were calculated as the sum of all items. Statements like *‘I will be able to achieve most of the goals that I set for myself’* reflect an individual’s confidence in their ability to complete tasks. Meanwhile, other statements, such as *‘Compared to other people, I can do most tasks very well’*, assess how individuals perceive their overall competence and self-worth. Overall, if one receives a score between 8 to 16 this is considered to be a low self-efficacy sum score, a score between 24 to 32 is a normal/moderate self-efficacy one and sum scores close to 40 indicate high self-efficacy (Chen et al., 2001; Gobeille et al., 2024). Chen et al. (2001) presented the findings of three studies introducing GSES and how it related to previous scales measuring self-efficacy and self-esteem. Firstly, Chen et al. demonstrated that the GSES has high content validity (Chen et al., 2001, p.77). Also, in all three studies reported in the paper, the reliability of the GSES was good (study 1 *α* = 0.85, study 2 *α* = 0.86 and study 3 *α* = 0.85). In the present study, the total scale reported a Cronbach’s *α* coefficient of 0.93.

### Anxiety

The state-trait anxiety inventory (STAI; Spielberger et al., 1983) was used to assess participants’ level of anxiety. The scale has 20 items measuring state anxiety with a four-point Likert scale (where 1 represents ‘not at all’ and 4 represents ‘very much so’) and 20 items with a four-point Likert scale assessing trait anxiety (where 1 represents ‘almost never’ and 4 ‘almost always’). Statements measuring state anxiety include *‘I feel calm’,* while statements assessing trait anxiety include *‘I lack self-confidence’.* The sums of scores from all items were calculated to generate a total anxiety score. The total range of anxiety indicates that if one receives a score between 40 and 79, they experience low anxiety, 80 to 99 moderate levels of anxiety and between 100 and 160 high anxiety with clinical concerns (Julian, 2011; Kayikcioglu et al., 2017). For the subscales, 45–80 is considered to be high anxiety. Substantial evidence supports the scale’s construct and concurrent validity (Spielberger, 1989). Spielberger et al. (1983) reported studies conducted to evaluate the scale’s test–retest reliability, which is reported as good (*α* = 0.65 and *α* = 0.75 after 2 months). In the present study, the analyses were carried out with the total scale scores, and these reported a Cronbach’s *α* coefficient of 0.96.

### Self-compassion

The self-compassion scale (SCS) was used to assess how adults with dyslexia act towards themselves under challenging circumstances (Neff, 2003a). The scale has 26 items with a five-point Likert scale (where 1 is ‘strongly disagree’ and 5 is ‘strongly agree’) and six sub-scales: self-kindness, self-judgement, common humanity, isolation, mindfulness and over identification. Scores were calculated for each sub-scale as a sum and mean. For the total score, a mean was calculated for the positive aspects self-kindness, common humanity and mindfulness and reverse scores for negative aspects self-judgement, isolation and over-identification. For example, a positively framed item is, ‘*When something upsets me, I try to keep my emotions in balance’*, while a negatively framed item is, *‘I can be a bit cold-hearted toward myself when I am experiencing suffering’.* Self-compassion is considered low when someone receives a score between 1 and 2.4, moderate between 2.5 and 3.4 and high self-compassion when it is between 3.5 and 5 (Neff, 2003b, 2016a). SCS is widely used in self-compassion research (MacBeth & Gumley, 2012; Neff, 2016b, 2020). Moreover, the original publication (Neff, 2003a) presented findings from three studies demonstrating the scale’s robust construct and discriminant validity. In the same publication, they demonstrated that SCS had high internal reliability (Cronbach’s *α* = 0.92) and test–retest reliability (Cronbach’s *α* = 0.93). In the present study, the total scale reported a Cronbach’s *α* coefficient of 0.89.

### Procedure

The University’s Ethics Committee approved the study. Respondents were asked to complete an online survey of approximately fifteen to 20 min on the JISC Online Surveys website (http://onlinesurveys.ac.uk/). Due to the nature of dyslexia as a SpLD causing reading difficulties, participants were offered a narrated version of the study upon request. Each participant answered demographic questions to understand whether self-compassion is more effective in certain life circumstances in mediating the negative emotional effects of dyslexia. The four scales assessing self-compassion, self-esteem, self-efficacy and anxiety were completed afterwards.

### Data analytic strategy

First, using correlation analysis, the relationship between dyslexia’s emotional outcomes was explored. To understand the potential impact of dyslexia on oneself, each self-compassion subscale and an overall score were included independently. This addressed an existing limited understanding of the influence of different facets of self-compassion (MacBeth & Gumley, 2012).

Self-compassion facets were explored as mediators between self-efficacy, self-esteem and anxiety by testing simple mediation models. These models test ‘*how*’ and ‘*why*’ facets of self-compassion account for the association between self-perception and anxiety in adults with dyslexia (Preacher & Hayes, 2004). This allows for an in-depth examination of the relationships between aspects of the self, which might prove valuable for incorporating compassion-focused elements within SEN provisions, including SpLD interventions.

The *total effect* of self-perception on anxiety was derived from a *direct effect* (weight of path *c*) of self-perception on anxiety and mediated effect (*indirect effect*; weight of path *ab*) of self-perception on anxiety through the hypothesised mediator (SCS total score or subscale scores of SCS). Weight path* a* signifies the *direct effect* of self-perception on self-compassion, and weight path *b* signifies the *direct effect* of self-compassion on anxiety while removing the influence of the effect of self-perception and any control variables. PROCESS v4.3 in SPSS was used to conduct the analysis. This tool allows bootstrapping to minimise the chances of type I and type II error while testing the indirect effects (Hayes, 2013). Confidence intervals (CI) for indirect effects were produced at 95% point estimates.

## The results

### Descriptive statistics

Scores were normally distributed for all variables (skewness < 2; kurtosis < 1). Descriptive statistics for the four scales are presented in Table 2. In summary, scores for the total self-compassion (SCS) and self-efficacy (GSES) scales are moderate, and scores for the STAI anxiety scale are higher than average in comparison to each scale’s expected (average) score, according to the manual (see ‘Materials’ section). In addition, scores were lower than the average proposed by the manual for the positive subscales of the SCS (self-kindness and mindfulness but not for common humanity), but this is not the case for the negative subscales, which indicate higher scores in this category (self-judgement, over-identification and isolation). As such, participants appear less compassionate towards themselves than they are hard on themselves. The scores for the self-esteem scale were below the normal range (average scores according to the scores given in the manual). The relationships between the variables were analysed by conducting a one-tailed Pearson’s correlation to investigate the first research question.

**Table 2:** Descriptive statistics

*Variable*	*M*		*SD*
SCS self-kindness (max. = 5)		2.63	0.85
SCS self-judgement (max. = 5)		3.69	0.89
SCS mindfulness (max. = 5)		2.87	0.84
SCS over-identification max. = 5)		3.65	0.95
SCS common-humanity (max. = 5)		3.17	0.70
SCS isolation (max. = 5)		3.63	1.02
SCS total (max. = 5)		3.27	0.44
Self-efficacy (max. = 40)		26.19	7.2
Self-esteem (max. = 30)		14.37	5.36
Trait anxiety (max. = 80)		53.4	11.8
State anxiety (max. = 80)		50.4	12.6
Anxiety (max. = 160)		104.07	23.51

*SCS *self-compassion scale (Neff, 2003a, 2003b); *M* mean; *SD* standard deviation; Max maximum. Scores are representative of the whole sample (N = 100)

### Correlation analysis

Positive and moderate correlations were found between scores for the STAI anxiety scale and negative facets of SCS (self-judgement, isolation and over-identification), and moderate negative correlations with positive aspects of SCS (mindfulness, self-kindness, and common humanity), as well as with the total score for SCS (see Table 3). Therefore, it seemed that as participants’ anxiety increased, self-compassion decreased. A reverse trend was observed for the SES self-esteem and GSES self-efficacy scores in relation to SCS: as self-esteem and self-efficacy scores increased, SCS scores also increased. Weak and moderate positive correlations between self-esteem and self-efficacy and positive facets of SCS were found. On the other hand, there was a weak and moderate negative correlation between self-esteem and self-efficacy scores and negative facets of SCS. In addition, self-efficacy and self-esteem scores were positively and moderately correlated with each other, while both self-esteem and self-efficacy scores were negatively and moderately correlated with STAI anxiety scale scores.

**Table 3:** Correlation analysis

Variables	1	2	3	4	5	6	7	8	9	10
(1) SCS self-kindness	-									
(2) SCS self-judgement	− 0.49***	-								
(3) SCS mindfulness	0.74***	− 0.32***	-							
(4) SCS over-identification	− 0.41***	0.72***	− 0.33***	-						
(5) SCS common-humanity	0.38***	− 0.02	0.55***	0.03	-					
(6) SCS isolation	− 0.34***	0.62***	− 0.28**	0.65***	0.12	-				
(7) Total SCS	0.47***	− 0.90***	0.35***	− 0.90***	− 0.04	− 0.83***	-			
(8) Self-esteem	0.57***	− 0.74***	0.41***	− 0.64***	0.09	− 0.64***	0.76***	-		
(9) Self-efficacy	0.38***	− 0.38***	0.39***	− 0.35***	0.06	− 0.32***	0.40***	0.58***	-	
(10) Anxiety	− 0.59***	0.65***	− 0.50***	0.69***	− 14	0.59***	− 0.73***	− 0.78***	− 0.57***	-

*p < 0.05, **p < 0.01, ***p ≤ 0.001

Demographic characteristics (age, diagnosis, age of diagnosis, gender, education and first language) were included in Pearson’s correlation analysis to ascertain whether any control variables should be included in further analysis. Because there were no significant correlations between the demographic variables and the scale scores for SCS, SES, GSES and STAI (all *p* > 0.05); the demographic variables were not included in further analyses. Ethnicity was positively associated with GSES scores (*r* = 0.35, *p* < − 0.001) and STAI anxiety scores (*r* = 0.29, *p* = 0.003) and non-significantly with SES (*r* = 0.17, *p* > 0.05) and SCS scores (*r* = − 0.19, *p* > 0.05). However, due to the homogeneity of ethnicity (with 82% White), this was not treated as a control variable either.

#### Mediation analysis

No multicollinearity issue was found between SES and GSES scores, and as such, these were treated as independent predictors within the mediation paths. The collinearity diagnosis indicated, however, potential issues for the facets of SCS self-compassion scores, as eigenvalues were close to 0 and some condition index values were greater than 15. Collinearity diagnosis indicated no multicollinearity issues after using *z*-scores. However, results of further analysis indicated the same pattern of findings when using means or *z*-scores, and consequently, results based on the means are reported.

**Fig. 1:**
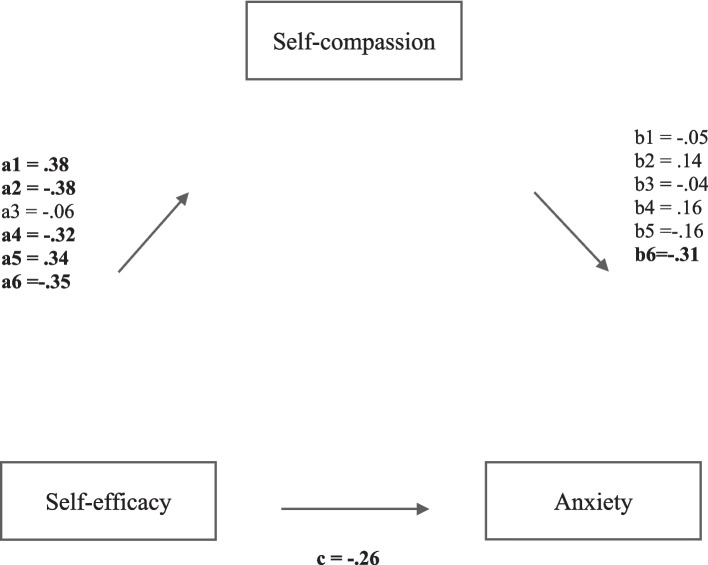
Multiple mediation analysis of facets of self-compassion on the relationship between self-efficacy and anxiety*.* a1, b1 refer to self-kindness as mediator. a2, b2, refer to self-judgement as mediator. a3, b3 refer to common humanity as a mediator. a4, b4 refer to isolation as a mediator. a5, b5 refer to mindfulness as mediator. a6, b6 refer to over-identification as mediator. Path *a* is the effect of self-esteem on the proposed mediator, path *b* is the effect of the proposed mediator on anxiety, and path *c* is the direct effect of self-esteem on anxiety. Mediation results are presented based on 5000 bootstrap resamples and 95% CI, significant pathways are denoted in bold

**Fig. 2:**
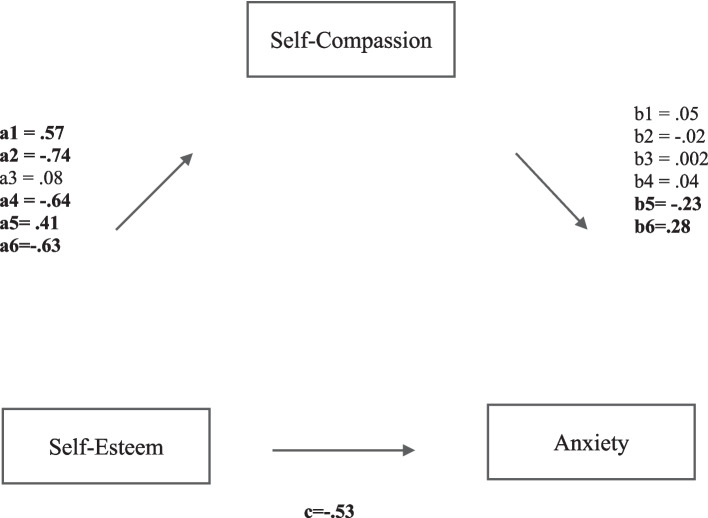
Multiple mediation analysis of facets of self-compassion on the relationship between self-esteem and anxiety. a1, b1 refer to self-kindness as mediator. a2, b2, refer to self-judgement as mediator. a3, b3 refer to common humanity as a mediator. a4, b4 refer to isolation as a mediator. a5, b5 refer to mindfulness as mediator. a6, b6 refer to over-identification as mediator. Path *a* is the effect of self-esteem on the proposed mediator, path *b* is the effect of the proposed mediator on anxiety, and path *c* is the direct effect of self-esteem on anxiety. Mediation results are presented based on 5,000 bootstrap resamples and 95% CI, significant pathways are denoted with bold

Initially, a simple mediation analysis was run using PROCESS model 4 (Hayes, 2013) in SPSS, with self-efficacy and self-esteem, respectively, as predictor variables (*X*_1,2_), self-compassion as the mediator (*M*) and anxiety as the outcome variable (*Y*). Mediation results are presented in Table 4 and Figs. 1 and 2. SCS total scores were employed to test the hypothesis stating that self-compassion (*M*) would significantly mediate the effects of self-esteem and self-efficacy (*X*_1,2_) on anxiety (*Y*).

**Table 4:** Mediation results based on 5000 bootstrap resamples

Predictor	Mediator	Total effect	Direct effect	Path a	Path b	Indirect effect	Effect size
Self-efficacy	Self-compassion total	− 0.55***	− 0.31***	0.40***	− 0.60***	** − 0.16 [− 0.25, − 0.07] †**	** − 0.24**
	Self-kindness		− 0.26***	0.38***	− 0.05	− 0.01 [− 0.06, 0.03]	− 0.02
	Self-judgement			− 0.38***	0.14	− 0.04 [− 0.09, 0.01]	− 0.06
	Common humanity			0.06	− 0.04	− 0.001 [− 0.02, 0.02]	− 0.002
	Isolation			− 0.32**	0.16	− 0.03 [− 0.08, 0.01]	− 0.05
	Mindfulness			0.34***	− 0.16	− 0.03[− 0.11, 0.01]	− 0.05
	Over-identification			− 0.35***	− 0.31***	** − 0.07 [− 0.15, − 0.02] †**	** − 0.11**
Self-esteem	Self-compassion total	− 0.87***	− 0.54***	0.76***	− 0.31***	** − 0.26 [− 0.46, − 0.10] †**	** − 0.24**
	Self-kindness		− 0.53***	0.57***	0.05	0.03 [− 0.1, 0.15]	0.03
	Self-judgement			− 0.74***	− 0.02	0.01 [− 15, 0.17]	0.01
	Common humanity			0.08	0.002	0.002[− 0.02, 0.03]	0.002
	Isolation			− 0.64***	0.04	− 0.02[− 0.15, 0.08]	− 0.02
	Mindfulness			0.41***	− 0.23***	** − 0.10 [− 0.22, − 0.01] †**	** − 0.09**
	Over-identification			− 0.63***	0.28***	** − 0.19 [− 0.34, − 0.06] †**	** − 0.17**

Adjusted coefficients with 95% CI. *p < 0.05, **p < 0.01, ***p ≤ 0.001. † Indicates a 95% confidence interval that does not include 0. Anxiety was the outcome

Firstly, using self-esteem as a predictor variable, the overall model was significant, *F*(2, 97) = 94.2*, p* < 0.001, explaining 66% of the variance in anxiety (*R*^2^ =. 66). The total effect of self-esteem on anxiety was significant, *β* = − 0.87, SE = 0.06, *t*(100) = − 12.6, *p* < 0.001. When controlling for the mediator, the direct effect of self-esteem on anxiety was reduced but remained significant, *β* = − 0.54, SE = 0.10, *t*(100) = − 5.9, *p* < 0.001, indicating partial mediation.

The indirect effect of self-esteem on anxiety through self-compassion was significant, *β* = − 0.24, SE = 0.08, with a 95% confidence interval (CI) of [− 0.42, − 0.09]. The mediation effect is significant since the CI does not include zero. Additionally, self-esteem significantly predicted self-compassion, *β* = 0.76, SE = 0.24, *t*(100) = 11.7, *p* < 0.001, and self-compassion significantly predicted anxiety, *β* = − 0.31, SE = 0.06, *t*(100) = − 3.42, *p* < − 0.001.

Next, using self-efficacy as a predictor variable, the overall model was significant, *F*(2, 97) = 78.6*, p* < 0.001, explaining 62% of the variance in anxiety (*R*^2^ = 0.62). The total effect of self-efficacy on anxiety was significant, *β* = − 0.55, SE = 0.05, *t*(100) = − 6.6, *p* < 0.001. When controlling for the mediator, the direct effect of self-efficacy on anxiety was reduced but remained significant, *β* = − 0.31, SE = 0.04, *t*(100) = − 4.5, *p* < 0.001, indicating partial mediation. The indirect effect of self-efficacy on anxiety through self-compassion was significant, *β* = − 0.24, SE = 0.046, with a 95% confidence interval (CI) of [− 0.36, − 0.13]. The mediation effect is significant since the CI does not include zero. Additionally, self-efficacy significantly predicted self-compassion, *β* = 0.40, SE = 0.08, *t*(100) = 4.4, *p* < 0.001, and self-compassion significantly predicted anxiety, *β* = − 0.60, SE = 0.05, *t*(100) = − 8.8, *p* < 0.001.

To test the third hypothesis, facets of self-compassion were analysed as independent mediators in a multiple mediation analysis to examine whether these mediated the relationship between self-esteem and self-efficacy (*X*_1,2_) and anxiety (*Y*) (using PROCESS model 4 (Hayes, 2013). The results showed different effects when self-esteem and self-efficacy, respectively, were included as predictors. Although self-esteem had a greater total effect on anxiety (*R*^2^ = 0.70, *F*(7,92) = 32.1, *p* < 0.0001, *β* = − 0.53) than self-efficacy (*R*^2^ = 0.66, *F*(7,92) = 26.15, *p* < 0.0001, *β* = − 0.26), the only sub-component of self-compassion that was a significant mediator in the relationship between self-efficacy and anxiety was over-identification (*p* < 0.001*)*, while isolation approached significance (*p* < 0*.*06). In addition, only mindfulness and over-identification were significant mediators between self-esteem and anxiety (*p* < 0.02 and *p* < 0.002*,* respectively).

Results showed that self-compassion as a total score was a more effective mediator than individual subscale scores in the relationship of both self-esteem and self-efficacy (predictors) and anxiety (outcome).

For the mediation analyses specified above in this section, it is argued that the difference between the total and direct effect of self-compassion on anxiety differs from zero. High self-esteem and self-efficacy were associated with self-compassion, which was linked to lower anxiety. It can also be claimed that not all sub-components of self-compassion have the same effect as mediators; thus, their effects differ depending on the predictor. As we included in our analyses the total anxiety score rather than the facets of state and trait anxiety, we also conducted a mediation analysis including as predictor variables state or trait anxiety with outcome variables self-esteem or self-efficacy and mediator self-compassion. The indirect effect on trait anxiety with predictors of self-esteem or self-efficacy was significant, and the effect sizes were higher than when the total anxiety score was considered as an outcome variable (ES = − 0.32 and ES = − 0.27, respectively). Similarly, the indirect effect on state anxiety with predictor self-efficacy was significant, and the effect size was lower than when the total anxiety score was considered as the outcome variable (ES = − 0.19). Finally, the indirect effect was not significant, and self-compassion was not a significant predictor of state anxiety; these results suggest that self-esteem influences state anxiety directly rather than through self-compassion, providing no support for mediation (ES = − 0.13). This outcome is further unpicked later in the ‘Discussion’.

## Discussion

Dyslexia was previously associated with negative emotional outcomes, including low self-esteem and self-efficacy and high anxiety (Burden, 2008; de Beer et al., 2014; Gibby-Leversuch et al., 2019; Livingston et al., 2018). Based on studies carried out with non-dyslexic populations, self-compassion was identified as a potential mediator for the relationship between these emotional outcomes (Dupasquier et al., 2020; Fong & Loi, 2016; MacBeth & Gumley, 2012; Neff, 2003b; Zhang et al., 2016). Therefore, a gap in the literature was identified in understanding the role of self-compassion in adults with dyslexia, which the current study aimed to fill.

Before testing the hypotheses and answering the research questions, indices of emotional characteristics were considered to understand the participants’ emotional state. These participants presented with self-esteem levels slightly below the thresholds that indicate concern for low emotions, which means that the sample in the study had a relatively low average self-esteem (see, for example, García et al., 2019). The scores for self-efficacy were in the normal (average) range, indicating that the sample was moderate in self-efficacy (Chen et al., 2001; Gobeille et al., 2024). Moreover, the anxiety scores were concerning, indicating high levels of anxiety, even potentially clinical levels of anxiety (Julian, 2011; Kayikcioglu et al., 2017). As for self-compassion, the overall scale suggested participants scored in the moderate levels of self-compassion, but when each sub-scale was considered, most indicated low or high self-compassion (depending on if it is a positive or negative fascet); only common humanity scores indicated moderate levels (Neff, 2003b, 2016a). Hence, the sample seemed to experience some emotional difficulties.

The first research question involved examining the relationships between self-compassion, self-esteem, self-efficacy and anxiety. Consistent with past research with non-dyslexic participants (Dupasquier et al., 2020; Fong & Loi, 2016; Neff, 2003a; Zhang et al., 2016), self-esteem, self-efficacy and anxiety were all significantly associated with self-compassion, supporting the hypothesis. However, self-compassion was more strongly related to self-esteem and anxiety than self-efficacy, indicating that it could have a more substantial effect on this relationship.

Moreover, the different subscale scores for the self-compassion measure correlated significantly with each other, as well as with the overall score for self-compassion and self-esteem, self-efficacy and anxiety, except in the case of the subscale common humanity. Common humanity was significantly and positively associated with other positive subscales of self-compassion (self-kindness and mindfulness) but not with negative subscales, the overall score for self-compassion or the other scales. According to self-compassion theory (Neff, 2003a), this could suggest that seeing one’s experiences with dyslexia as part of the greater human condition could be linked with the participants’ kindness towards self and mindfulness; yet, this view is not associated with how much individuals critique themselves and overidentify with negative feelings. Hence, it could be argued that having an awareness of the commonality of dyslexia alone does not protect individuals from self-judgement and negative feelings. The results of the study of Zhang et al. (2016) indicated a similar pattern, which indicates that common humanity might have a more distinct role than other subscales of the SCS self-compassion measure.

The second hypothesis, which examined the role of self-compassion in moderating the effects of self-efficacy and self-esteem on anxiety, was also supported. Self-compassion appears to have a similar role in adults with dyslexia as in neurotypical samples (Neff, 2003a; Neff, 2016b; Neff, 2020; Fong & Loi, 2016). The indirect effect of self-compassion was, however, greater in the relationship between self-esteem and anxiety than in the relationship between self-efficacy and anxiety. Nevertheless, the effect sizes for the two mediations did not differ, indicating that self-compassion is similarly effective in mediating the relationship between different concepts within the umbrella of self-perception and anxiety. Also, these effect sizes (− 0.24) were similar to those reported in pre-existing literature (e.g., Bergey et al., 2015; Carroll & Iles, 2006; MacBeth & Gumley, 2012). Nevertheless, studies need to include more measures and larger samples to explore how self-compassion is associated with other self-related elements and, henceforth, achieve a more holistic insight into dyslexia. Moreover, it is important to remember that this is a correlational design, so longitudinal studies could be valuable for further exploring these associations.

MacBeth and Gumley’s (2012) suggestion regarding examining the role of the different subscales of self-compassion was addressed with multiple mediation analyses. Results indicated that the SCS self-compassion subscale scores were less effective mediators than the overall score. This supports the argument of Neff (2020) that self-compassion is a more robust variable when an overall score is used rather than subscale scores. Nevertheless, the findings showed that different sub-components of self-compassion have distinct roles within the explored relationships, which has not been reported before. Thus, the successful mediators were not the same in the relationship between self-esteem and anxiety as in the relationship between self-efficacy and anxiety. The facet of self-compassion that was an effective mediator in the relationship between self-efficacy and anxiety, and self-esteem and anxiety was over-identification. This is not unexpected as we explored a sample of individuals who identify themselves through the disability. Interestingly, mindfulness mediated the relationship between self-esteem and anxiety, obviously potentially operating as a protective factor against feelings of low self-worth and feelings of helplessness.

Contrary to the general belief that being more kind towards self, less judgemental, seeing one’s condition as part of the larger human experience, and not feeling isolated have an indirect effect on how negative emotions affect wellbeing (MacBeth & Gumley, 2012; Neff, 2003a), the findings from the current study contest this view regarding self-efficacy and self-esteem on anxiety. Only being mindful about and not over-identifying with one’s negative experiences of self-esteem seems to play a significant role in reducing the anxiety caused by low self-esteem, whilst self-efficacy has to do only with less identifying with the condition. Consequently, the effect of low self-esteem on anxiety could potentially be more challenging to remediate than the effect of low self-efficacy on anxiety.

Pointing out that over-identifying with difficulties or feeling isolated (although it approached significance) can have a more detrimental impact on individuals than being mindful, having a kind understanding of one’s experiences and viewing these as a part of the human condition could have a positive impact. The findings suggest that it is more important to be mindful of not acting negatively towards self, rather than simply having very positive attitudes. Nevertheless, as this has not been studied before in this population, caution needs to be exercised when generalising the findings, particularly given the relatively small sample size and the correlational design.

It is clear that to identify the needs of people with dyslexia, a detailed understanding of their emotional experiences is required. Psychological outcomes of dyslexia could impact well-being negatively (Burden, 2008; de Beer et al., 2014; Livingston et al. 2018; Gibby-Leversuch et al., 2019); hence, specialists are advised to include emotional considerations to respond to the large population with dyslexia with diverse psychological needs (Carroll & Iles, 2006; Doyle, 2020; Elftorp & Hearne, 2020 Niolaki et al., 2020). One such model that takes this into consideration is the biopsychosocial model (BPS; Edgel, 1977). Considering self-compassion theory (Neff, 2003a) within the biopsychosocial model (Edgel, 1977), the findings from the current study could help further the current understanding of the psychological and emotional processes experienced by individuals with dyslexia**.** The insight gathered from the study demonstrates that self-compassion as a way of relating to the self could potentially play a significant role in the well-being of adults with dyslexia. Consequently, incorporating self-compassion in practice when working with these individuals might lead to identifying their needs or strengths more efficiently and holistically (Doyle, 2020; Elftorp & Hearne, 2020). For those found to struggle with self-efficacy and/or self-esteem, increasing self-compassion could be a positive factor in dealing with their difficulties. Thus, using self-compassion as a strength consciously might lead to better psychological coping (Niolaki et al., 2020).

Further, Engel (1977) suggested that psychological well-being (or illbeing) is directly linked with social experiences and experiences of having a disability. It could be that being self-compassionate is associated with physiological reactions and impacts the social life of individuals. Whilst the current study only included psychological measures linked to emotions, adopting a more holistic view of dyslexia and examining the potential somatic consequences (such as high blood pressure, diabetes and autoimmune illnesses) could lead to better conceptualisation of the SpLD and understanding of the needs of those who are in search of support.

We aimed to establish a new conceptualisation for understanding the relationship between self-perception (self-esteem and self-efficacy), self-compassion and anxiety. This relationship has not been studied previously in a dyslexic sample but was consistent with predictions based on pre-existing research. Hence, the uniqueness of the study is furthering existing findings that report self-compassion as a potential mediator for negative emotions (Dupasquier et al., 2020; Fong & Loi, 2016; Zhang et al., 2016) and also for adults with dyslexia. This paper can help individuals with dyslexia, families and employers make informed choices concerning how they interact with people with dyslexia. Thus, the exploratory component of the study is highly valuable for a more holistic understanding of this relationship, as the findings revealed that not all elements of self-compassion have the same indirect effect on the association of self-perception and anxiety. This further pointed to a need to provide tailored individual emotional support for people with dyslexia.

Interestingly, we also explored whether self-compassion mediates the relationship between self-esteem, self-efficacy and the two subcomponents of anxiety (trait and state anxiety). Our findings suggest that trait anxiety plays a more central role in shaping these relationships, while state anxiety appears to have a lesser influence. This may be because participants were not placed in stressful situations, unlike in previous studies (for example, in the study of Carroll & Iles, 2006, the participants were asked to complete a literacy test). The lack of literacy measures and the fact that some individuals self-identified as dyslexic present additional challenges when interpreting the results.

As with all research, this study is not free from limitations. Firstly, global scales for self-perception and anxiety were included. Previous literature emphasised that dyslexia has a distinct effect on dimensions of self-esteem and anxiety, greater affecting academic and literacy domains (Bergey et al., 2015; Carroll & Iles, 2006; Elgendi et al., 2021; Jordan et al., 2014). Investigating these variations and implementing a similar design would potentially add depth to the current understanding of the relationship between self-related variables in adults with dyslexia.

Another limitation is that it is unknown how much of the measured emotional outcomes are directly caused by the experiences with dyslexia and how much other variables intervene in the studied relationships. The measures the study investigated relied upon assuming negative outcomes of dyslexia based on previous findings (Burden, 2008; de Beer et al., 2014; Livingston et al., 2018; Gibby-Leversuch et al., 2019). To address this limitation, a scale measuring the experience with dyslexia could be included in further studies adopting a similar method, for example, the scale developed by Nalavany et al. (2011) measuring the emotional experience with dyslexia. Including other potential co-variates could also be useful, for example, co-morbidity, to research the role of self-compassion in adults with dyslexia who also experience other difficulties. Additionally, based on the biopsychosocial model (Engel, 1977), self-compassion could be linked to biological and social dimensions. Whilst the current study was able to aid understanding of the psychological experiences of adults with dyslexia, researching how this is linked to physiological outcomes such as cortisol levels or social outcomes such as work participation would help achieve a more holistic conceptualisation. This could be achieved by employing experimental designs.

Further, the participants varied in age and educational background but were fairly homogenous in cultural background, including ethnicity and first language spoken. As in Nalavany et al.’s (2017) study, these factors were not influential in the analysis of the present study. However, the current results indicated that ethnicity might have a relationship with the studied variables, but conclusions could not be drawn from this sample. This is something that could be explored in future research. Moreover, it should be noted that although participants had the opportunity to request a narration of the study, no participant used this setting. Being able to complete the study independently could potentially indicate that the sample does not have severe issues in reading. Incorporating a measure of severity would enhance the study (e.g., a reading fluency test), along with increasing the sample size, as the current sample may have been too small to account for the number of moderator variables included. We had individuals who were both diagnosed as dyslexics and a smaller proportion of self-diagnosed dyslexics (*N* = 22); although, there was not a significant difference in the critical variables (i.e., self-esteem, self-efficacy, self-compassion and anxiety), still a measure of literacy or a cognitive one can be an additional criterion for identification of needs. However, we are also aware that with dyslexic adults, sometimes, literacy challenges can be resolved or compensated, and only cognitive challenges such as with memory, organisation or processing would remain (see, for example, a discussion on this in the latest UK definition of dyslexia, Kirby et al., 2024). Lastly, the design is correlational, so one cannot claim causal associations between the explored variables.

It is also important to discuss access to work and the benefits of coaching and mindfulness in mitigating psychological and emotional challenges. These benefits are potentially evident in our study of the positive effect of knowing to be mindful and having healthy self-compassion, which can make individuals less fearful, judgmental, worrisome and over-ruminating. However, on the other hand, not being able to compartmentalise difficulties and over-identifying with those can have a detrimental effect on self-esteem and increase the dyslexic person’s anxiety. Although there are not many quantitative studies to compare the findings with, a qualitative paper that followed successful and less successful adults with dyslexia from childhood to adulthood also supports our findings and the need to be able to look at the positive elements and traits rather than the challenges that dyslexia imposes (Goldberg et al., 2003). We hope that our study will be able to add a little bit more evidence of this.

## Conclusions

Dyslexia affects a high proportion of the population in the UK (Snowling, 2019). Adults with dyslexia are an under-investigated population at risk of developing negative emotional outcomes caused by their difficulties (Burden, 2008; de Beer et al., 2014; Gibby-Leversuch et al., 2019; Livingston et al., 2018). To further the understanding of their psychological experiences, the current study investigated the role of self-compassion in adults with dyslexia by testing two hypotheses and further addressing an exploratory question about the role of different facets of self-compassion (self-kindness, self-judgement, common humanity, isolation, mindfulness and over identification). First, known negative outcomes of dyslexia (low self-esteem, low self-efficacy and high anxiety) were correlated with self-compassion. Second, self-compassion was a significant mediator in the relationship between self-esteem and self-efficacy, respectively, and anxiety. Hence, the first and the second research questions were answered, and the expectations were supported. Finally, the study revealed that sub-components of self-compassion have distinct roles in mediating the effects of self-esteem and self-efficacy on anxiety. That is to say, the findings from the exploratory question added depth to the current understanding of the role of self-compassion. Nevertheless, as a hidden disability, dyslexia seems to have effects on the self; yet, it is not well documented and requires further investigation. Additionally, enhancing self-companionship may help mitigate some of the adverse psychological and emotional effects associated with dyslexia. As a way forward, training studies can examine how coping strategies for adult dyslexics may help to improve self-compassion and respectively improve self-esteem and self-esteem and reduce anxiety.
